# “Will it Work as Well on Zoom?” A Natural Experiment During the Covid-19 Pandemic of Delivering Parenting Groups Via Video Conferencing or in Person

**DOI:** 10.1007/s10826-022-02398-8

**Published:** 2022-08-08

**Authors:** Livia van Leuven, Maria Lalouni, Martin Forster

**Affiliations:** 1 Department of Clinical Neuroscience, Division of Psychology, Karolinska Institutet, Nobels väg 9, 171 65 Solna, Sweden; 2 Department of Clinical Neuroscience, Division of Neuro, Karolinska Institutet, Nobels väg 9, 171 65 Solna, Sweden

**Keywords:** Parent-training, Universal prevention, Digital intervention, Mixed-methods, Covid-19

## Abstract

While rates of child maltreatment increased during the Covid-19-pandemic, face-to-face interventions to support families got difficult to carry out due to restrictions. Meanwhile, many services do not have access to parenting programs designed for digital or remote delivery. A solution employed by some services was to use video conferencing (VC) to deliver their regular parenting programs. This study examined the effectiveness of the universal group-based parenting program ABC offered through VC instead of on-site meetings during the pandemic. Pre and post measurements were collected from 469 parents participating in either 1) ABC with VC meetings only, 2) on-site meetings only, or 3) blended – a combination of VC and on-site sessions. In addition, 74 group leaders completed a survey about their experiences of VC groups. Analyses showed general improvements in parent practices and child conduct over time, but no differences in effectiveness depending on the format of the parent group (VC, blended, or on-site). Qualitative analyses of group leaders’ experiences revealed four key-themes pertaining to both challenges (e.g., concerns about parents’ ability to benefit and learn parenting skills) and benefits (e.g., reaching parents who would not have been able to attend physical meetings) of VC groups. Overall, this study showed no significant differences in outcomes between the VC, blended, or on-site format of delivery. There are however limitations of this trial, and results should be considered preliminary. Effectiveness and potential negative consequences of replacing interventions intended to be delivered on-site with VC alternatives need to be further investigated in future trials.

## Highlights

- While the pandemic put families at strain, on-site support programs got difficult to deliver due to pandemic restrictions.
- In Sweden, clinics offered the universal parenting program ABC through video conferencing (ABC-VC).
- This study examined outcomes of ABC conducted by VC versus on-site, and how group leaders experienced ABC-VC.
- Improvements in parenting skills and children’s behaviors were similar for parents receiving ABC on-site and by VC.
- Video conferencing can be a way to reach more families. Meanwhile, some parents may benefit more from an on-site group.

## Background

Mental illness is a world-wide problem associated with great suffering to individuals (Polanczyk et al., 2015) and large costs to society (Seabury et al., 2019). For many who suffer from mental illness in adulthood, the disorder debut during childhood (Jones, 2013). In the early development of children’s health or ill health, parents play a key role. The quality of the parent-child relationship has long-standing impacts on the child’s health (e.g., Stewart-Brown et al., 2005). Interventions focusing on the parent-child relationship and on parental behavior are therefore suggested as a key to overcoming the large societal burden that mental illness presents today (Yap et al., 2016).

Universal parenting programs can be implemented to prevent mental health and behavioral problems by reducing the occurrence of risk factors and enhancing protective factors around the child. The focus of such programs is to teach parents strategies to handle challenging situations and to improve parent-child relationships. Parenting programs have been evaluated in many trials and are disseminated across the world (Leijten et al., 2019). One program that is widely adopted in Sweden is the universal parenting program the *All Children in Focus* (ABC) program. ABC has been evaluated in an RCT with positive effects on child health and parent practices (Ulfsdotter et al., 2014), and is today employed by clinics all over the country. Hundreds of parents participate in ABC every year. The current study is part of a larger ongoing nation-wide study where data on ABC is collected continuously to evaluate its dissemination and effects in regular services.

When the Covid-19-pandemic started, conducting parenting programs was suddenly difficult. Many programs, including ABC, are in the format of group meetings which were not possible to carry out due to pandemic restrictions. A demand for other ways to organize parenting programs therefore arose, not the least since the pandemic and its consequences, such as parental stress, loneliness, job loss, and income instability, increased rates of child maltreatment and abuse (Lawson et al., 2020; Rodriguez et al., 2020). One solution to this problem was to offer group parenting programs through remote delivery, instead of on-site meetings (Sullivan et al., 2021).

There is a growing evidence base for the effectiveness of delivering parenting programs through digital solutions (Baumel et al., 2016; Bausback & Bunge, 2021; Thongseiratch et al., 2020). Even when modes of delivery were compared directly in the same study, a digital version was found to be non-inferior to the on-site version of a parenting program (Prinz et al., 2022). However, most studies of remotely delivered parenting programs have relied on self-directed content (e.g., internet-based programs with text, videos, and online-based exercises) with therapist support through text messaging or telephone. Thus, most studies investigate the effects of programs that have been specifically developed for digital delivery (Baumel et al., 2016; Bausback & Bunge, 2021; Thongseiratch et al., 2020). However, at least in Sweden, the access to such programs in regular services is low. Instead, group-based parent training with on-site meetings are offered. During the Covid-19 pandemic, many services started to provide their regular parenting programs through video conferencing (VC), in lack of access to programs developed for digital or remote use. Despite the necessity for many services to transfer to VC tools during the pandemic (Sullivan et al., 2021), the evidence base for this type of remote delivery is rather limited. Only a couple studies with few participants have investigated the use of VC to deliver group-based parenting programs (Canário et al., 2021; Fogler et al., 2020; Reese et al., 2012; Reese et al., 2015; Xie et al., 2013). While the reported effects on symptoms and fidelity have been promising, the evidence is still uncertain.

The practice to offer a group based parenting programs through VC instead of on-site may be viewed as a variation in implementation rather than a novel type of program – i.e., it is not a new intervention, but an existing intervention implemented in a new way. And in the case it is conducted spontaneously by practitioners, rather than planned by program developers, it is important to identify if it should be considered as program drift and a threat to fidelity, or if it is a constructive innovation and necessary adaptation (Fixsen et al., 2005). During the Covid-19 pandemic, it can clearly be argued that transferring existing programs to VC delivery was a necessary adaptation, given that the alternative often was no intervention at all. On that note, in one of the most common frameworks in implementation science (RE-AIM), the *reach* of an intervention is defined as one of five core dimensions that is important to include in evaluations of interventions (Glasgow et al., 1999). In other words, it does not matter how effective an intervention is, if it does not reach the intended target group. Potential reach is of course one of the general benefits of digital delivery of interventions (Kazdin, 2019: Sullivan et al., 2021; Vigerland et al., 2016). A prerequisite is however the second dimension of RE-AIM: *efficacy*. The efficacy of delivering parenting programs through VC is still uncertain, given the limited number of studies specifically addressing that issue.

When testing a new way of delivering an intervention, the last three dimensions of the RE-AIM model are equally important to consider. To what extent is it *adopted* by the relevant services and to what degree of fidelity is it *implemented*? And will the new way of delivery be *maintained* over time? Besides addressing these issues with quantitative methodology, it is also important to include qualitative methods to generate hypotheses of possible barriers and facilitators of implementation through different modes of delivery. The inclusion of qualitative methods in research can be especially helpful in novel and time-pressed contexts, like the Covid-19 pandemic (Vindrola-Padros et al., 2020).

## The Current Study

The current study is part of a larger ongoing nation-wide project evaluating ABC in regular services, in which more than 100 sites are involved with thousands of parents that submit assessments before and after they have taken part of ABC. No results from this project have yet been published. The current study consists of a subset of the data from the project, collected during the pandemic. When the pandemic started in early 2020, the owners of the copyright of ABC (City of Stockholm) allowed all sites to use VC as an alternative to on-site group meetings. Since local restrictions varied, some clinics offered ABC through VC group meetings only, some used a blend of VC and on-site meetings, while some employed regular on-site meetings only. This natural experiment offered an opportunity to compare these different modes of delivery in the current study. Besides using quantitative data from the larger ongoing study, qualitative data on group leaders’ experiences of delivering ABC through VC were also collected as a complement.

The aim of this mixed methods (MM) study was to answer the following research questions:

1. Does mode of delivery of the ABC-program affect parenting and child outcomes?
2. How do group leaders experience the delivery of ABC through VC?

## Methods

### Study Design and Procedures

To develop an understanding of several aspects of ABC-VC, this study used a convergent MM design – to collect both quantitative and qualitative data, analyze the data separately, and then compare and merge the findings. MM is based on the assumption that one method insufficiently captures all aspects of a topic. Thus, combining qualitative and quantitative data contribute to a richer analysis (Creswell & Creswell, 2018).

To compare ABC provided by VC versus on-site, quantitative data was obtained from an ongoing project (not yet published) where data from ABC groups in regular care all over Sweden have been collected since 2018. While the larger study aims to evaluate reach and predictors of program effects during the large-scale dissemination of ABC in Sweden, the current study aims to evaluate different ways of delivering ABC during the pandemic period. The sample of the current study consisted of parents participating in the project from the start of the Covid-19-pandemic to the Spring of 2021. Sweden had rather few nationally implemented restrictions during the pandemic which gave each municipality the mandate to decide upon local restrictions. Therefore, some sites offered ABC by VC (*n* = 37), some by on-site meetings (*n* = 50), and some sites had blended groups (*n* = 26). Among the 50 sites that had groups with on-site meetings, 28 also organized groups with VC meetings while the remaining 22 did not use VC at all. Parents included in this article were divided into three groups based on the format of the parent group they participated in: 1) VC – ABC consisting of VC meetings *only*, 2) Blended – ABC conducted with a combination of VC and on-site meetings, and 3) On-site – ABC with on-site meetings, i.e., its original format.

Clinics offering ABC invited parents who took part in ABC to participate in the research and thereby answer a survey at the first (T1) and the last meeting of the parent group (T2), respectively. Group leaders informed parents about the project orally and in writing. Parents who gave written consent to participate completed the questionnaire (5–10 min) digitally or on paper. Group leaders were encouraged to ask parents to complete T2 from home in cases where parents dropped out of ABC. Parents declining participation in the study participated in the ABC-groups according to the same procedure as the research participants.

Qualitative data was group leaders’ responses to open-ended questions pertaining to their experiences of ABC-VC. The questions were included in a survey sent out in the summer of 2021 to group leaders who had arranged at least one group with VC meetings.

### Participants

Participants in the study were parents and group leaders arranging ABC groups. To cover the pandemic period, parents responding at T1 from the beginning of February 2020 to the end of May 2021 were included. Ten parents were excluded because group format was not stated (i.e. on-site, blended, or VC) in the post-survey and we could not retrieve information about which kind of group they had participated in from the group leader. In total, 469 parents living in 59 Swedish municipalities were included. Of these, 241 (51%) participated in an on-site group, 87 (19%) in a blended group, and 141 (30%) in a VC group. Because the information of the parent group format (VC, blended, or on-site) was obtained at T2, the sample only includes parents answering the survey at both T1 and T2. ABC is a universal parenting program for parents with children aged 3–12 years. To explore its effects in real-life conditions, no inclusion- or exclusion criteria were employed for the study. There were no criteria for assignment to format (VC, on-site, or blended); the sites decided on what format to deliver ABC through, which was driven by pandemic restrictions. For demographics of the families, see Table 1.

**Table 1:** Characteristics and demographics of parents *N (%)* or *M (SD)*

	Total *N* = 461	On-site *n* = 237	Blended *n* = 84	VC *n* = 138	X^2^	*p*
*Parent characteristics*						
Proportion of mothers	346 (75.1)	178 (75.1)	56 (66.7)	112 (81.2)	6.89	0.14
Parental educational level						
Elementary school	10 (2.2)	8 (3.4)	0 (0.0)	2 (1.45)	3.81	0.15
High school/vocational school	167 (36.2)	101 (42.6)	27 (32.1)	39 (28.3)	**8.56**	**0.01**
University	264 (57.3)	117 (49.4)	55 (65.5)	92 (66.7)	**13.62**	**0.01**
Other	18 (3.9)	11 (4.6)	2 (2.4)	5 (3.6)	0.89	0.64
Parent born in Sweden	392 (85.0)	205 (86.5)	68 (81.4)	119 (86.2)	1.64	0.44
*Characteristics of focal child*						
Age	5.74 (2.6)	5.75 (2.7)	6.18 (2.6)	5.46 (2.4)	4.84	0.09
Proportion of girls	184 (39.9)	99 (41.8)	32 (38.1)	53 (38.4)	0.91	0.63
Number of siblings	1.14 (0.8)	1.18 (0.7)	1.21 (0.81)	1.02 (0.7)	**7.09**	**0.03**

Statistically significant differences between groups are presented in bold; Kruskal-Wallis Test was used to calculate numerical data (age and siblings of child) and Chi2 for the remaining (categorical) outcomes

At least 228 group leaders, mostly working in pairs, led the groups. Among these, 36 had at least one group in two formats (e.g., one on-site group and one VC) and five had one or more groups in all three formats. There might have been more group leaders involved that we were not able to identify; parents sometimes only reported the name of one and the recommendation is to lead a group together with a colleague. The VC group survey (see below) was sent out to 103 group leaders, who had led at least one VC or blended group. In total, *n* = 74 (71%) responded. Known reasons for non-responding were email addresses not working (*n* = 6) and on leave of absence at the time the survey was sent out (*n* = 10).

### Measurements

#### Questionnaires to parents

Questions about parenting practices and child problem behaviors were assessed at T1 and T2. The instruments were chosen based on feedback from a feasibility assessment prior to the study, showing that it was critical to keep the number of items to a minimum; a longer survey was first tested, resulting in high attrition. To avoid selection bias (e.g., only highly motivated and educated parents responding to the questionnaires), each instrument contains very few items. Since no questionnaires with such few items could be identified, the research group constructed the instruments, based on established instruments and clinical experience.

Parent practices were measured with six questions about how often different parent behaviors had occurred the last two weeks. Parents responded on a 7-point likert scale from 1 (‘never’) to 7 (‘many times a day’). Four questions assessed positive parenting practices (PPP). More specifically, whether the parent had played or done something nice with the child, praised the child, prepared the child, and talked calmly with the child when angry. E.g., “How often have you praised your child in the last two weeks?”. These questions were inspired by the questionnaire Parenting Young Children (PARYC; McEachern et al. (2011)). Inter-item correlations for PPP ranged from 0.20 to 0.56 (*M* = 0.33) at T1 and 0.19 to 0.49 (*M* = 0.28) at T2, which is acceptable (Clark & Watson, 1995). Cronbach’s alpha was α = 0.66 at T1 and α = 0.60 at T2. The remaining two questions pertained to negative parent practices (NPP; nag or yell at the child), e.g., “How often have you yelled at your child in the last two weeks?” (inter-item *r* = 0.51 at T1, α = 0.67, and *r* = 0.58, α = 0.72 at T2).

Child problem behaviors was measured by the CPB-scale, a brief instrument that is used in the parent project of the current study. It consists of four questions assessing the impact of conduct problems and disruptive behaviors in different life areas over the last two weeks; “Has the child been fighting or disturbing in the last two weeks so it’s been a problem in school or preschool?”, and equivalent for ‘home’, ‘with friends’, and ‘leisure activities’. Items are rated on a likert-scale from 0 (“Not at all”) to 3 (“Very much”). Inter-item *r* = 0.21 – 0.62 (*M* = 0.34), α = 0.65 at T1, and *r* = 0.15 – 0.42 (*M* = 0.26), α = 0.56 at T2. The CPB-scale has been psychometrically evaluated in the parent project to the current study, which will be presented in an upcoming publication (van Leuven et al., 2022). In that analysis, ratings from 211 parents on the CPB-scale and the Eyberg Child Behavior Inventory (ECBI; Eyberg & Pincus, 1999) was related to diagnostic status of their child as well as independent clinical assessments of child conduct problems (Clinical severity rating). The correlation between CPB and ECBI was moderate, but the ability to discriminate children with diagnosis was stronger for the CPB-scale than the ECBI.

Parental satisfaction and experience of improvement after ABC was measured at T2 with a six-item questionnaire (Inter-item *r* = 0.13–0.58, *M* = 0.32; α = 0.74). Five of the questions were taken from the 10-item Therapy Attitude Inventory (TAI; Brestan, Jacobs, Rayfield, & Eyberg, 1999). TAI focuses on parents’ perceptions of having developed skills during an intervention and their general satisfaction with the help they have received. For example, items like ‘My general feeling about the program I participated in…’ and ‘The major behavior problems that my child presented at home before the program started are at this time…’. Questions were responded to on a 5-point likert scale, e.g., 1: ‘considerably worse’ – 5: ‘greatly improved’. One item was added that was constructed by the research group; ‘How well do you think the group leaders conducted the meetings?’.

The T1 questionnaire also contained questions about background and demographics. The T2 survey included a question about how often parents had completed homework between the group meetings rated on a 5-point likert scale from 1 (‘Single occasions or not at all’) to 5 (‘Several times a day’). At T2 parents were also asked if the parent group had consisted of VC or on-site sessions, or both.

#### Questions to group leaders

Two separate surveys were sent to group leaders. The first survey (‘Background survey’) was sent prior to the current study to all group leaders working with ABC in Sweden. The second survey (‘VC group survey’) was constructed for this study and sent out to group leaders who held a VC or blended group during the Covid-19-pandemic (Spring 2020 – Spring 2021) and for whom an email address could be found.

The background survey contained questions about group leaders’ previous background (education, workplace, and years of professional experience). The VC group survey contained questions concerning how they had conducted ABC-VC (how many VC groups, what VC platform, if the sessions had been in groups of parents or individually), and their experiences of holding digital groups. All questions were quantitatively measured except for two open-ended questions pertaining to the group leaders’ view on possible advantages and dis-advantages of having parenting groups by VC. The quantitative questions of experiences of VC groups pertained to parents’ completion of homework assignments (slider scale from 0: ‘less homework completion’ to 10 ‘as much as an on-site group’) and parental attendance or dropout (likert scale from 1: ‘a lot less drop outs’ to 5: ‘a lot more drop outs’) during VC groups *compared to* on-site groups. The survey also included a question of whether the VC format had influenced the group leaders’ possibilities to guide parents to other services if they needed more help (e.g. social services or psychiatry). The VC group survey was constructed by the research team for the purpose of this study. Selection and construction of individual items were discussed and decided upon within the team.

#### Intervention

ABC is a manualized, universal parenting program for parents with children aged 3–12 years. The program is based on social learning theory and attachment theory and the aim is to promote parental competence and child development, by targeting the parent-child relationship. ABC has been shown to improve parent self-efficacy and parent-reported child health and development (Ulfsdotter et al., 2014). The original version of the program (on-site) consists of four 2.5 h sessions held approximately every second week in groups of about 10 parents. The parents receive handouts with the session content and completes homework between meetings. Each session focuses on different themes: 1) Showing love, 2) Being there, 3) Showing the way, and 4) Picking your battles, see Table 2 for a description of the modules. The group leaders initiate discussions, show videos and use role-play to emphasize key messages. For more details about the ABC program, see Lindberg et al., (2013).

**Table 2:** Content of the modules in the ABC program

Module	Content
1. Showing love	In the first module parental warmth and love is presented as a fundament for the relationship between parents and children. Modeling and parental attention are introduced as important ways in which children learn from parents. Focusing on what works is taught as a way to get into virtuous circles, with less conflicts in the family.
2. Being there	In the second module, spending time together in the family is encouraged. Also, child-directed play is introduced, in which parents encourage, reflect, and follow their child’s lead. The module also teaches functional analysis to understand children’s challenging behaviors and prevention of conflicts by using routines, preparations, positive expectations, child involvement, and encouragements.
3. Showing the way	In the third module, the disadvantage of using anger as a parenting strategy is emphasized and the parents learn ways to calm themselves in challenging situations. This includes identifying triggering situations, raising awareness of their own physiological responses to them, and learning strategies to reduce their anger or stress by for example taking a break. The parents also work with identification and reduction of overall life stressors.
4. Picking your battles	The fourth module teaches parents about the importance of reducing nagging, complaints, and unnecessary reprimands. The parents are also taught a strategy to handle their children’s discontent in situations when they need to pick the battle: Validate the child’s feelings, explain briefly why the child can’t have his or her own way, and distract the child by giving another option.

ABC was developed 2010–2012 by the Social Services in Stockholm. The development was conducted in cooperation with parents, group leaders of parenting programs, researchers, and representatives from municipalities and city districts in Stockholm. The development and the initial evaluation of ABC was financed by the Swedish government as a part of a national effort to promote parenting in Sweden. The implementation of ABC started already during the development phase 2010 and the program is now widely implemented in most municipalities and regions in Sweden. It has not yet been implemented in any other country. The City of Stockholm is the owner of the copyright of ABC.

When delivered by VC, group leaders were encouraged to conduct ABC just as the on-site version of the program except for that the meetings were by VC. The owners of the copyright of ABC (the City of Stockholm) sent out information to group leaders that they were allowed to deliver ABC through VC, due to the pandemic, and that the program should still be delivered according to the manual. I.e., to conduct the same lectures, discussions, exercises, and role plays, but via video. The information to group leaders also contained some advice for how to deliver VC groups, e.g., to have more short breaks, to send parents their material before the meetings, that parents should sit undisturbed, and advice on how to distribute the word in the group. No other adaptation of the program was done to facilitate remote delivery. We have limited knowledge of potential local adaptations by the group leaders leading the groups.

#### Statistical analyses

Analyses were conducted with R statistical software version 1.4.1106 (RStudio Team, 2021) and Jamovi version 1.6.23.0. Baseline differences between the groups on demographic variables and parent ratings were analyzed with Chi 2-tests, One-Way ANOVA and, for numerical variables where the assumption of normality was violated, Kruskal-Wallis and Dunn’s tests. The change over time on parent ratings of parent practices and child conduct were analyzed with Two-Way Mixed ANOVA, and satisfaction with the program and homework completion with One-Way ANOVA. Due to indications of CPB not being normally distributed, the variable’s 10% trimmed means was also analyzed with a robust within-between Mixed ANOVA employing the WRS2 package (Mair & Wilcox, 2020). Analyses were conducted first with three (on-site, blended, and VC) and then with two (on-site and VC) levels on the grouping variable. When both parents in a family answered questionnaires, one parent’s data was randomly selected to be included in the analyses. No á priori power calculation was conducted because we did not know in advance that VC groups would be part of the ongoing project. The sample size in the study implied a statistical power of 80% to detect a small effect size (*d* = 0.20) with alpha = 0.05. The sample size was adjusted in the power calculation since the groups were unequal in size. The total sample size was therefore set to 252, which represents the smallest group (blended = 84 participants) multiplied by 3 (Faul et al., 2009). Data obtained by group leaders are presented descriptively.

##### Missing data

There was 0.8% missing data in parent ratings. The majority of missing values was due to some parents skipping the question about talking calmly with their child due to them not having been angry during the last two weeks. To handle missing data points, the total score of each scale was estimated by computing an average of the items in each scale and multiplying with the number of items.

#### Qualitative analyses

To identify recurrent themes in group leaders’ responses to the open-ended questions, reflexive thematic analysis following the steps described by Braun and Clarke (2006; 2019) was carried out by the first author. The purpose of the analysis is to identify patterns (themes) in the data. Data was analyzed inductively and no specific theory was applied. Data was analyzed across questions (not separately for each question) to identify themes in the whole dataset. Due to limited amount of qualitative data, data was mainly coded on a semantic level i.e., identifying content and patterns without in-depth analyses of underlying patterns and concepts. In the first phase of analyzes, the data set was read and notes were taken. In the second phase, initial codes were generated to identify potentially interesting features of the data. If appropriate, a single statement was coded with several codes. Once all data had been coded, phase 3 began where codes were sorted into initial, candidate themes. Next (phase 4), themes were reviewed by reading the data for each theme and the data set in its whole and revising themes to ensure they coherently reflected patterns in the data. The themes were further refined in phase 5 by writing an analysis of each theme, identifying themes’ core features, and along the way refining themes and sub-themes. The essence of each theme was further developed in phase 6 (writing the report).

#### Data integration

We compared results using a side-by-side MM comparison, i.e., to interpret and compare quantitative and qualitative results in the discussion (Creswell & Creswell, 2018).

## Results

### Participant Characteristics

Demographics of parents and children are presented in Table 1. The number of siblings differed significantly between groups. Dunn’s test of pairwise comparisons showed that the difference was due to slightly more siblings in the on-site group compared to the VC (*p* < 0.05). Analyses also showed that more parents in the blended and VC groups had a university education than parents in the on-site groups, in which more parents’ highest education level was high school or vocational school (*p* < 0.01). Furthermore, One-Way ANOVA showed no differences between the three groups at baseline on the parent rated measures presented in Table 3 (PPP, NPP, and CPB; *p* > 0.05).

In total, 178 of the group leaders who led the groups responded to a survey about their previous education and experience. Common professional backgrounds were preschool teachers (*n* = 78), social workers (*n* = 57), teachers of children in need of special support (*n* = 57), nurses (*n* = 7), and psychologists (*n* = 5). Most common workplaces were Family centers – a type of agency where health care for children and mothers, social services, and open playgroups for children are located in the same place (*n* = 53), social services (*n* = 41), school (*n* = 36), and preschool (*n* = 32). Group leaders who held on-site groups only (*n* = 101 with available data) had 1–45 (*M* = 11, *SD* = 11) years of professional experience in providing support or treatment for parents, not statistically different from group leaders who held groups with VC meetings (*n* = 74; 1 – 43 years, *M* = 11, *SD* = 10). The group leaders had varying experience with ABC; on-site only-group leaders had experience in holding 1-22 (*M* = 4, *SD* = 4) groups each, which was less than group leaders who held groups containing VC meetings (1–42, *M* = 6, *SD* = 7 groups each; *p* < 0.05).

A significant difference between groups in follow up time from T1-T2 was found (*F* = 27.33, *p* < 0.001). Days from T1-T2 in the three conditions: On-site: *M* = 91, *SD* = 84; Blended: *M* = 102, *SD* = 74; VC: *M* = 42, *SD* = 21. The follow-up time was in general longer than the 60 days that is the length of ABC when offered strictly as advised. One reason that came to our knowledge was that some groups starting during Spring 2020 were set on pause due to Covid-19 and continued during the autumn.

### The Adjustment of ABC to a VC Format During the Covid-19-Pandemic

Group leaders had led 1–10 (*M* = 2.05, *SD* = 1.56) VC or blended ABC groups each. To arrange meetings, they had used Microsoft Teams (*n* = 61), Zoom (*n* = 8), Google Meet (*n* = 6), Skype (*n* = 4), Pexip (*n* = 1), and telephone (*n* = 1). Almost all of the group leaders (*n* = 72) reported that the majority of their digital ABC-meetings had been in groups (i.e., including parents from more than one family). The remaining two conducted the majority of their ABC-VC meetings individually (parents to children in the same family).

### Effects on Children and Parents of ABC-VC

Mean values, standard deviations (SD) and statistics for repeated measures ANOVA with three levels (on-site, VC, and blended) on the grouping variable are presented in Table 3. Not taking the format of the parent group into account, PPP improved significantly over time, while NPP and CPB were significantly reduced (*p* < 0.001 for all three measures). The change over time did not depend on the format of the parent group as shown by the lack of interaction effect. Results were the same for CPB employing robust ANOVA (main effect of group value = 0.24, *p* = 0.79; main effect of time value = 50.91, *p* < 0.001; interaction effect value = 1.06, *p* = 0.35). As also shown in Table 3, parents in the three conditions were equally satisfied with ABC. Analyses were also carried out with two levels on the grouping variable (VC versus on-site). The results were the same as for analyses including the blended groups (i.e., a significant effect of time at *p* < 0.001, no significant group nor group by time interaction effect). Regarding homework completion, there was a tendency (three level analysis: *p* = 0.090; two level analysis: *p* = 0.053) towards that parents in the VC condition completed homework slightly more than parents in the other conditions. Finally, since parents in the on-site groups had lower education levels, additional analyses were carried out for each outcome with education as a covariate. The analysis showed that controlling for educational level, the results remained the same with no significant differences between conditions on any of the outcome measures.

**Table 3:** Mean values (SD) and statistics at T1 and T2

	On-site			Blended			VC			Time × Group		Time		Group	
	T1	T2	*d*	T1	T2	*d*	T1	T2	*d*	F (2)	*p*	F (1)	*p*	F (2)	*p*
PPP^1^	16.3 (4.38)	17.2 (4.00)	0.22	16.0 (4.08)	17.3 (3.28)	0.35	16.8 (3.85)	18.1 (3.50)	0.34	0.56	0.57	35.83	<0.001	2.04	0.13
NPP	7.72 (2.45)	6.14 (2.11)	0.69	7.96 (2.51)	5.96 (1.89)	0.89	7.93 (2.55)	6.35 (2.02)	0.68	1.00	0.37	192.80	<0.001	0.53	0.59
CPB	2.49 (1.96)	1.70 (1.42)	0.44	2.30 (1.77)	1.64 (1.19)	0.43	2.28 (1.81)	1.72 (1.42)	0.34	0.90	0.41	68.58	<0.001	0.32	0.72
Sat^1^		26.22 (2.36)			26.49 (2.07)			25.82 (2.56)						2.29	0.10
HC		3.04 (1.03)			2.98 (1.11)			3.25 (1.04)						2.46	0.09

^1^An increased score at T2 indicated a change in a favorable direction. *PPP* Positive Parent Practices, *NPP* Negative Parent Practices, *CPB* Child Problem Behavior, *Sat* Satisfaction questionnaire, *HC* Homework Completion

### Group Leaders’ Experiences of VC Parent Groups – Quantitative Measures

Among the 74 group leaders who answered the survey about arranging ABC-VC, 63 answered the questions about parents’ homework completion and dropouts. The remaining 11 did not answer these questions because they had never conducted an on-site ABC-group. Regarding how much group leaders experienced that parents had completed homework assignments during VC groups compared to groups on-site (0: much less – 10: as much as a physical group), the average rating was 8.65 (*SD* = 1.83). Further, 24 group leaders reported that there had been *less* drop outs during digital parent groups, 20 reported that dropout rates had been about the same as during on-site groups, and 19 experienced it as more dropouts during VC groups.

Regarding how the group leaders thought that the VC format had influenced their possibilities to guide parents in need of more help, *n* = 4 experienced it as easier/better in the VC format, *n* = 29 as no difference, and *n* = 25 as more difficult. The remaining 16 group leaders reported that they could not answer the question.

### Group Leaders’ Experiences of VC Parent Groups – Qualitative Analyses

Qualitative analyses revealed four key themes: 1) More can participate, 2) Parents may attend and learn less, 3) Rethinking the group leader job, and 4) Less relationship building. Themes and sub-themes are presented below. The number of group leaders touching upon a theme or aspect of the analysis is presented in brackets.

#### More can participate

Group leaders experienced that a greater variety of parents, living in different life circumstances, can participate in VC than on-site groups. This advantage was mostly described regarding families’ life situation in general and not specifically concerning the pandemic circumstances.

##### When traveling takes too much time

Group leaders experienced that digitization increases the availability of support [40]. Groups can be easier to attend in a stressful daily life thus parents save time on logistical arrangements [30] such as not having to travel to a clinic; parents attended meetings from their workplace or from home [24]. Some experienced less dropouts in their VC groups [5]. Several others, although not expressing a general increase in attendance during VC groups, experienced that their digital groups had reached more parents than groups on-site [20]. There had been more variation in where the attending parents lived. Parents who live far away from clinics (e.g., in non-rural areas or cities where ABC is not held) can participate [8]. A few [5] wrote that parents had said that they only had the possibility to participate because ABC was offered remotely: “Parents said that they could now participate because it was digital. They commuted to work and lived in the outskirts of the municipality which made physical participation more difficult”.

##### No babysitter needed

It was also easier for some parents to participate because they did not need to arrange a babysitter [18]. More single parents [3] and both parents in the same family [7] were thus able to participate more frequently. A few wrote that children too small to be on their own could be together with parents during sessions [3].

##### Pandemic compatible

Some pointed out that an advantage was that parenting programs could be offered during the pandemic [4]. Parents could also participate despite them or their children being sick, which would have restricted them from attending an on-site group during the pandemic [7].

##### Safe to join from home

A final sub-theme was group leaders experiencing parents as safe and relaxed due to them sitting at home [5]: “Parents opened up and shared with each other very quickly. I experienced parents being emotionally safe when sitting at home”. A few pointed out that digital groups may attract parents who are not comfortable with joining a physical group [3]: “To participate and be more anonymous, maybe you had not dared to come to a physical group, regardless of the pandemic”.

#### Parents may attend and learn less

A second theme concerns that group leaders experienced a shift in how parents attended sessions in VC compared to on-site groups. Factors in the remote situation may influence the quality of parents’ attendance and concentration during sessions.

##### Not as present

The remote situation brought distractions to the environment where parents attended the meeting [9]. Parents sometimes did other things while attending meetings, e.g., were traveling from work. One group leader pointed out that difficulties arise when there is not enough space in the family’s house for the parent to sit undisturbed. Children being at home sometimes took focus from the program [6]. Technical problems also disturbed sessions [25], e.g., difficulties watching videos or presentations [8] and problems with internet connection [7]: “Technical problems take time from conversations. Some parents missed parts of discussions because of technical problems.” Five group leaders had also experienced more dropouts during their VC groups. One wrote that almost half of those who signed up did not participate. Two hypothesized that it might be easier for parents to drop out of VC groups.

##### Less discussions

It was also more difficult to start and keep discussions going during VC groups [19]. Parents discussed less [9] and discussions were less natural [11]. Parents talked one by one to a greater extent [3]. As one put it: “It is a step to ask for the word, the spontaneous may not come”. It may be more difficult for some parents (e.g., those who are shy) to say something during VC than physical groups [6].

#### Rethinking the group leader job

The VC format changed group leaders’ work situation and working conditions. New challenges arose which needed time and practice to handle.

##### Changed practicalities

The practical arrangements before, during, and after groups became different. Group leaders saved time when holding VC groups, e.g., on traveling and preparing before sessions [11]. Meanwhile, technical problems posed a new challenge [27]. To do a good job, group leaders need to practice to feel comfortable in using the technology [7].

##### Difficult to know if parents are active and understand

It was more difficult for group leaders to know if parents were following sessions and thus to give accurate support if they were not. Group leaders experienced it as difficult to perceive the mood in the group and of individual participants during the VC meetings [16]. It was more difficult to know how parents reacted to what was said and if they had understood. Some parents choose not to turn on their camera which made it particularly difficult to know if parents followed the session [4]. It was also difficult to know if parents completed the pre- and post-measurement [2].

##### Must work harder to reach out

Group leaders needed to work harder to keep conversations going and to demonstrate exercises during the VC groups. Some wrote that they needed to be more active in guiding conversations [11] and put more effort into keeping up the atmosphere [4]. Some experienced that it was more difficult to reach out because they could not use their body language in the same way as during on-site groups [3]. It was also more difficult to role-play to demonstrate examples of situations [5].

#### Less relationship building

Group leaders missed the social part of on-site groups. It was more difficult to build relationships when meeting through VC. The online groups focused mainly on the content of the program, while the social parts around the meetings were lost.

##### More difficult for parents to get to know each other

Attending a parent group can be an opportunity to get to know other parents. However, because parents interacted less in the VC groups it was more difficult for them to get to know each other [16]. One group leader thought that it would have been easier for parents to share experiences if they knew each other better. It was difficult to provide individual communication between parents. Parents did not have the chance to network and chat before and after sessions and in the breaks, as during an on-site group [15]. The atmosphere in the group was different during VC groups [6]. It can be easier to create an inclusive atmosphere and sense of community when meeting on-site [4].

##### Difficult for group leaders to get to know parents

Without these social interactions, it was also difficult for group leaders to get to know parents and build relationships with them [6]. One group leader wrote that because it takes longer to get to know parents it is more difficult to be supportive when someone gets emotional. Another group leader thought that they might not be able to guide parents to further help to the same extent when not knowing parents as well.

## Discussion

The current study aimed to evaluate different ways of delivering ABC during the Covid-19-pandemic. The first aim was to evaluate if mode of delivery (VC, blended, or on-site) would affect parenting and child outcomes. The analyses showed that parent practices improved and CBP decreased in all modes of delivery. There were no statistically significant differences in change over time between the three conditions. The second aim was to investigate the group leaders’ experience of delivering ABC through VC. Group leaders experienced both advantages and disadvantages of VC compared to on-site groups. Some (38%) experienced less dropouts during VC groups and some (30%) more. Half (50%) of the group leaders experienced no difference in their ability to guide parents in need of more help during VC than on-site groups while 41% experienced this as more difficult in VC groups. Qualitative analyses revealed four key themes. Group leaders experienced VC to offer a greater flexibility that makes it easier for parents to attend, but could also negatively impact learning during sessions. In addition, the digital format made it more difficult to build relationships within the groups. To hold VC groups also brought new challenges for the group leader to handle during sessions. These results suggest potential positive and negative consequences of using VC that should be taken into account in the implementation of parenting programs in regular practices.

The lack of differences between the study conditions in parenting and child outcomes is an important addition to the evidence base of using VC as delivery method of group-based parenting programs. The results are in accordance with, to our knowledge, the only previous controlled study of VC-delivered parenting programs (Xie et al., 2013), that also showed essentially no difference between the VC and on-site conditions. Our results are also in line with a couple of pre-post studies in which VC was used as way of delivery, where effect sizes were reported to be similar to other studies of on-site parenting programs (Canário et al., 2021; Reese et al., 2012; Reese et al., 2015). Even if the evidence of the effectiveness for parenting programs that have been developed for digital delivery (e.g., internet-based parenting programs) is fairly strong (Baumel et al., 2016; Bausback & Bunge, 2021; Thongseiratch et al., 2020), it is important to build evidence for the use of VC as delivery method of parenting programs designed for on-site group meetings. This is not the least imperative in times of crises or in settings that lack recourses, in which access to platforms and programs designed to be delivered digitally may be limited. VC-delivery gives services an opportunity to instantly offer their existing group based parenting programs through remote delivery. Since parenting programs is suggested as a key in preventing mental ill-health (e.g. Yap et al., 2016), the expansion of mental health services through novel models of delivery is crucial (Kazdin, 2019). In particular since rates of child abuse increased during the pandemic (Lawson et al., 2020; Rodriguez et al., 2020). Our results suggest that parenting programs such as ABC could be offered by VC under such circumstances.

In research and development of novel ways of intervention delivery, it is relevant to consider several aspects of implementation besides efficacy. In terms of the RE-AIM framework (Glasgow, 1999), the group leaders’ experience of using VC to deliver parenting programs offered some notable insights. First of all, several group leaders experienced that VC-delivery had an impact on *reach* of the parenting program, in the sense that a more diverse group of parents could participate. This is a common argument for the dissemination of digital interventions in general (Kazdin, 2019; Sullivan et al., 2021; Vigerland et al., 2016), and with the addition of VC to other digital delivery methods the reach could become even wider. On the other hand, the demographics of the included participants suggest that the reach of the digital offering may be narrower in regard to education and larger families, since the parents who choose to be part of ABC-VC on average had higher education, and parents in the on-site condition had more children than those in the VC condition.

The qualitative analysis did not offer any specific insights related to the third dimension of the RE-AIM framework (adoption), but the natural experiment in itself shed some light on the matter. Of the 113 clinics that participated in the study, 63 (56%) started to offer ABC through VC or blended format when that opportunity was allowed by owners of the copyright of ABC. The true proportion would probably have been higher if all clinics would have been prohibited to arrange on-site group meetings (i.e., no variation in local pandemic restrictions).

How will VC impact the adherence and quality of the delivery of parenting programs – “Will it work as well on Zoom?” That question was of central interest in the study and several conclusions from the quantitative and qualitative analysis concerns *implementation*, the fourth dimension of the RE-AIM model. First, according to the self-assessments, parents participating through VC reported at least as much homework completion as parents in on-site groups. The group leaders who used VC also experienced that the parents in VC-groups completed almost as many homework assignments as parents in regular on-site groups. In terms of completion rate, about as many group leaders reported that VC resulted in more dropout, as those who experienced a decrease in dropout compared to on-site delivery. One interpretation of this is that VC suits some groups of parents better than others, and a way to improve the general reach and attendance to parenting programs would be to offer VC as a complement to on-site meetings within the same service. That the dropout rate (on average) seemed to be unaffected by VC delivery was however encouraging, given that completion and retention has been expressed as one of the challenges with other methods of digital delivery in regular services. For example, in a recent meta-analysis of ICBT for depression and anxiety in routine care, only 30% of screened patients were offered ICBT. Of those, 73% started the program, and of those almost 40% dropped out (Etzelmueller et al., 2020).

Despite the encouraging results regarding attendance and homework completion, the group leaders also experienced challenges with VC in terms of implementation. Some group leaders experienced difficulties in guiding parents in need of more help as well as more dropouts compared to on-site groups. In addition, group leaders thought that parents’ ability to concentrate could be negatively impacted by the remote situation, due to e.g., distractions in the surroundings and technological problems. It was also more difficult for parents to get to know each other. One consequence could be that while group VC could suit some parents better than on-site groups, some could benefit less. Our qualitative analyses indicate that parents who fit better in an *on-site* group could be characterized by being in a greater need of social support or having problems concentrating, either due to individual difficulties or to family factors (e.g., living crowdedly). Significant predictors and moderators of parenting programs in previous research include factors that contribute to a more disadvantaged situation regarding e.g., socioeconomic status (e.g., Reyno & McGrath, 2006). It is possible that parents who experience more difficulties are also those in need of more social support, which can be more difficult to get in a VC group.

Group leaders experienced that the remote situation changed their own working conditions and the role of the group leader, which could have influenced fidelity to the manual and overall quality of ABC. Along with the perceived differences in parents’ abilities to benefit from ABC-VC, this could indicate that some adaptations of the program are needed. Some group leaders did not report as many challenges as others. It is possible that the variation could have been due to a varying degree of local adaptations of ABC when conducted by VC, that we have no knowledge of. Many also experienced that technological problems interrupted sessions and a number of group leaders did not feel comfortable in using the technology. Besides maintaining the quality of the program, addressing such issues can also be of importance regarding data confidentiality, which is a critical issue in digital health care delivery where sensitive information is continuously transferred. In addition, group leaders reported using many different softwares to deliver ABC, of which some are not high-security alternatives. When VC is employed in regular care, training in using the technology is needed as well as access to VC software that are easy to use, have functions to facilitate delivering the program with quality (e.g., to use whiteboard and have small group discussions), and secure enough to maintain confidentiality.

Taken together, our results indicate that VC can be a viable alternative to on-site meetings in terms of effectiveness. Meanwhile, there are both pros and cons of VC versus on-site groups that should be taken into account when planning a group and deciding what is the best fit for families. For families who are motivated to work independently, VC can be a good choice. Families needing more support could suit better in a physical group. When VC is used, clear guidelines for how to participate can be useful (e.g., how to create an undisturbed home-environment). Group leaders can also benefit from training in how to offer programs by VC (e.g., in handling technological problems, data security, and facilitating online communication between parents).

This study has several strengths regarding external validity. Families were recruited through regular services and no exclusion criteria were employed. We therefore expect the sample to be representative for the parents who actually attend ABC in Sweden. In addition, the study captures the experiences of group leaders who work in regular services with ABC, some of whom have for years. Since the pandemic was ongoing during the entire study period we also expect all groups to be exposed to about the same circumstances. Also, the combination of quantitative and qualitative methods applied in this study contribute with different perspectives and therefore a more comprehensive analysis.

Conclusions about effectiveness from this study are limited by the study design; we have only tested for superiority, we have limited knowledge of parents who dropped out or chose not to participate, and parents were not randomized to conditions. The lack of randomization does imply risk for selection bias, but it may have been countered by the fact that neither the parents nor group leaders were able to self-select condition. Instead, the decision of whether ABC was conducted by VC or on-site was mainly based on restrictions implemented at the municipal or regional level. To establish the effectiveness of ABC-VC compared to ABC on-site, a non-inferiority design would have been a superior research design. The achieved statistical power was however insufficient for a non-inferiority study, given that the non-inferiority margin would be rather small (universal parenting programs generally produce small to medium effects).

Another limitation concerns the lack of validated measurements. However, the experiences from the larger study that data was collected from had called for a short survey with questions relevant to the content of ABC to facilitate receiving responses from as many parents as possible, in order to achieve a representative sample. A further limitation concerns the lack of data on the fidelity of the intervention and the extent to which groups were adjusted to comply with the pandemic situation and VC format. The group leaders’ experiences indicate that some adjustments of ABC was most likely inevitable to cope with practical challenges and to better fit the needs of some parents. To have data on if, and if so how, group leaders adapted the program would have been useful. An adjustment that did come to our knowledge was that some groups starting Spring 2020 were set on pause and continued during the autumn. This, and possibly other reasons, influenced the follow up time to be longer than expected in two of the groups (on-site and blended). However, the aim was to evaluate ABC in the circumstances of the pandemic. Several of these limitations were therefore an inevitable part of this trial.

Limitations regarding the qualitative part of the study concerns the limited amount of qualitative data per group leader. However, we wanted the perspective of many group leaders and with a questionnaire we reached more than we would have had the opportunity to interview.

In future research, the effectiveness of VC as a delivery method of parenting programs needs to be investigated in RCT’s. Preferably, by comparing VC to golden standard face-to-face options using a non-inferiority design. Research on factors influencing parents’ ability to benefit from or participate in VC-delivered parenting programs is also needed. Our findings suggest that parents’ ability to benefit could be influenced by individual or family level factors which need further investigation in quantitative trails. Guidelines for how to tailor support to families and to decide whether a family should join on-site or by VC are also needed.

## Conclusions

The lack of differences in outcomes between study conditions supports that ABC could be offered through VC. This study also reports that group leaders experienced challenges in conducting VC groups concerning concentration during meetings, relationship building within groups, and for the group leader to work digitally. Meanwhile, they also experienced major benefits; it could be easier for parents to participate in VC meetings compared to on-site. Overall, this study points to VC being an accessible and effective way of delivering interventions. The rapid adoption of VC by the included clinics in the study also speaks to the usefulness of the technique in times of crises, when many services suddenly need to find alternatives to their regular on-site delivery.
